# Esketamine nasal spray shows greater improvement in health-related quality of life over 32 weeks versus quetiapine extended release in patients with treatment resistant depression

**DOI:** 10.1192/j.eurpsy.2024.749

**Published:** 2024-08-27

**Authors:** A. H. Young, B. T. Baune, N. Cardoner, R. Frey, T. Ito, Y. Kambarov, A. Lacerda, B. Rive, C. von Holt, A. J. Oliveira-Maia

**Affiliations:** ^1^Department of Psychological Medicine, Institute of Psychiatry, Psychology and Neuroscience, King’s College London, London; ^2^South London and Maudsley NHS Foundation Trust, Bethlem Royal Hospital, Beckenham, United Kingdom; ^3^Department of Psychiatry, University of Münster, Münster, Germany; ^4^Department of Psychiatry, The University of Melbourne, Melbourne, Australia; ^5^Hospital de la Santa Creu i Sant Pau Universitat Autònoma de Barcelona (UAB), Barcelona, Spain; ^6^Department of Psychiatry and Psychotherapy, Medical University of Vienna, Vienna, Austria; ^7^Janssen EMEA, High Wycombe, United Kingdom; ^8^Janssen EMEA, Beerse, Belgium; ^9^Laboratório Interdisciplinar de Neurociências Clínicas, Universidade Federal de São Paulo, São Paulo, Brazil; ^10^Janssen EMEA, Paris, France; ^11^Janssen EMEA, Neuss, Germany; ^12^ Champalimaud Research and Clinical Centre, Champalimaud Foundation; ^13^NOVA Medical School, Faculdade de Ciências Médicas, NMS, FCM, Universidade NOVA de Lisboa, Lisbon, Portugal

## Abstract

**Introduction:**

In ESCAPE-TRD esketamine nasal spray (ESK-NS) significantly increased the probability of achieving remission at Week (Wk) 8 and being relapse‑free through Wk32 after remission at Wk8 versus (vs) quetiapine extended release (Q-XR) in patients (pts) with treatment resistant depression (TRD) (Reif *et al.* DGPPN 2022; P-01-04). We report ESK-NS vs Q-XR effects on pt-reported health-related quality of life (HRQoL) over 32 wks.

**Objectives:**

Evaluate pt-reported HRQoL using the generic 36-item Short-Form Health Survey version 2 (SF-36v2, 4-wk recall, 2009 US population norms) in ESCAPE-TRD.

**Methods:**

ESCAPE‑TRD (NCT04338321) was a randomised phase IIIb trial comparing the efficacy of ESK-NS vs Q-XR, both alongside an ongoing selective serotonin/serotonin-norepinephrine reuptake inhibitor, in pts with TRD. SF-36v2 was assessed every 4 wks (on-treatment and retrieved dropout visits). Domain scores and change from baseline (CfB) were analysed using a mixed model for repeated measures (MMRM; observed cases) adjusted for age, prior treatment failures, baseline score. Higher scores indicate better HRQoL. P values were not adjusted for multiple testing.

**Results:**

336 and 340 pts were randomised to ESK-NS and Q-XR. Baseline domain scores were below general population norms and lowest in Role Emotional, Mental Health and Social Functioning (** Figure 1A**). All scores improved to Wk32 in both arms (** Figure 1B**). At Wk4, CfB was significantly higher (better HRQoL) with ESK-NS vs Q-XR across domains (all p<0.01). At Wk8, CfB was significantly higher with ESK-NS vs Q-XR across all domains (p<0.05) except Bodily Pain and Role Physical. At Wk32, CfB was significantly higher with ESK-NS vs Q-XR for Mental Health (p=0.014), Role Emotional (p=0.001), Role Physical (p=0.046) and Social Functioning (p=0.006); a trend of numerical advantage was seen for all other domains (** Figure 2**).

**Image:**

**Figure:**
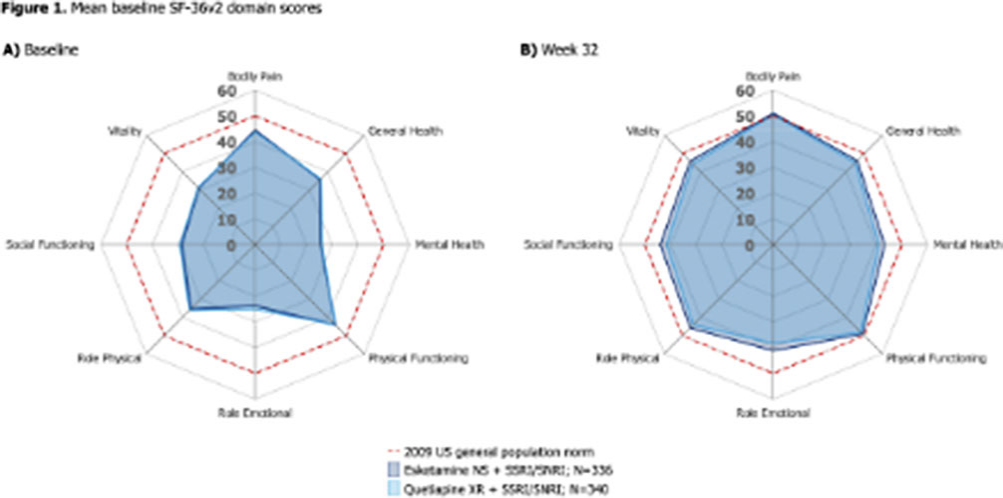

**Image 2:**

**Figure:**
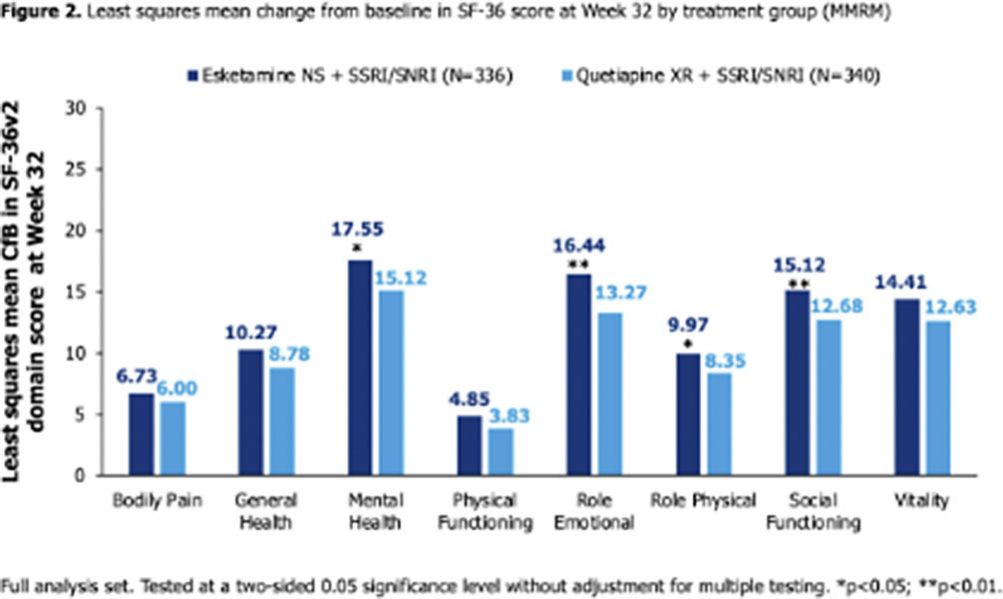

**Conclusions:**

In addition to the superior clinical benefits provided by ESK-NS vs Q-XR in ESCAPE-TRD, pts receiving ESK-NS experienced significantly greater improvements in HRQoL vs Q-XR over 32 wks.

**Acknowledgements:**

We thank the patients who participated. Study funding: Janssen, medical writing: Costello Medical, UK.

**Disclosure of Interest:**

A. Young Grant / Research support from: Received grants from Janssen; independent research funded by the National Institute for Health Research (NIHR) Biomedical Research Centre at South London and Maudsley NHS Foundation Trust and King’s College London; the views expressed are those of the authors and not necessarily those of the NHS, the NIHR, or the Department of Health, Consultant of: Received consulting fees from Allegan, AstraZeneca, Bionomics, Eli Lilly, Janssen, Johnson & Johnson, LivaNova, Lundbeck, Servier, and Sumitomo Dainippon Pharma and Sunovion, Speakers bureau of: Received speaker’s honoraria from Allegan, AstraZeneca, Bionomics, Eli Lilly, Janssen, Johnson & Johnson, LivaNova, Lundbeck, Servier, and Sumitomo Dainippon Pharma and Sunovion, B. Baune Grant / Research support from: Received research grants from private industries or non-profit funds from AstraZeneca, Lundbeck, and Sanofi-Synthélabo; received research grants from the BMBF and BMG Germany, the DFG, Germany, the National Health and Medical Research Council, Australia, and Horizon Europe 2021; received research grants from the Fay Fuller Foundation, and James & Diana Ramsay Foundation, Adelaide, Consultant of: Received consulting fees for roles with the National Health and Medical Research Council, Australia; received honoraria from Angelini, AstraZeneca, Biogen, BMS, Boehringer Ingelheim, Johnson & Johnson, LivaNova, Lundbeck, Otsuka, Pfizer, Roche, Servier, Sumitomo Dainippon Pharma and Sunovion, and Wyeth; served on advisory boards for Biogen, Boehringer-Ingelheim, Janssen-Cilag, LivaNova, Lundbeck, Novartis, and Otsuka, N. Cardoner Grant / Research support from: Received research grants from the Ministry of Health, Ministry of Science and Innovation (CIBERSAM), and the Strategic Plan for Research and Innovation in Health (PERIS) for the period 2016–2020, as well as from Marato TV3 and Recercaixa, Consultant of: Served on advisory boards for Angelini, Esteve, Janssen, Lundbeck, Novartis, Pfizer and Viatris, Speakers bureau of: Received speaker’s honoraria from Angelini, Esteve, Janssen, Lundbeck, Novartis, Pfizer and Viatris, R. Frey Grant / Research support from: Received travel fees from Janssen and LivaNova; received grants or contracts from Alkermes (Principal Investigator), Janssen (Principal Investigator), LivaNova (Principal Investigator) and Medizinisch‑Wissenschaftlicher Fonds des Bürgermeisters von Wien (academic study), Consultant of: Received consulting fees from Boehringer Ingelheim and Janssen, Speakers bureau of: Received speaker’s honoraria from Janssen and Lundbeck, T. Ito Shareolder of: Johnson & Johnson, Employee of: Janssen, Y. Kambarov Employee of: Janssen, A. Lacerda Grant / Research support from: Received grants from Azidus, Biophytis, Boehringer-Ingelheim, Cellavita, Celltrion, CNPq, Eli Lilly, EOM, FAPESP, Genova, IQVIA, Janssen, Nordisk, Novartis, Novo, Parexel and PPD, Consultant of: Received consulting fees from Aché, Apsen, Biogen, Boehringer-Ingelheim, Cristalia, Daiichi, Eurofarma, Sankyo, EMS, Janssen, Libbs, LivaNova, Lundbeck, Sanofi and Torrent, Speakers bureau of: Received speaker’s honoraria from Aché, Apsen, Biogen, Boehringer-Ingelheim, Cristalia, Daiichi, Eurofarma, Sankyo, EMS, Janssen, Libbs, LivaNova, Lundbeck, Sanofi and Torrent, B. Rive Employee of: Janssen, C. von Holt Shareolder of: Johnson & Johnson, Employee of: Janssen, A. Oliveira-Maia Grant / Research support from: Received grants from Compass Pathways, Ltd., Janssen, and Schuhfried GmBH; investigator‑driven research funded by Fundação para Ciência e Tecnologia (PTDC/SAU-NUT/3507/2021; PTDC/MED-NEU/1552/2021; PTDC/MED‑NEU/31331/2017), Fundação para Ciência e Tecnologia and FEDER (PTDC/MED-NEU/30845/2017_LISBOA-01-0145-FEDER-030845; PTDC/MEC-PSQ/30302/2017_LISBOA-01-0145-FEDER-30302), the European Research Council (ERC-2020-STG-Grant 950357), the European Commission Horizon 2020 Research and Innovation program (H2020‑SC1‑ 2017‑CNECT‑2‑777167‑ΒΟUNCE; H2020‑SC1‑DTH‑2019‑875358‑FAITH), and the European Joint Programme in Rare Diseases (Joint Translational Call 2019) through Fundação para Ciência e Tecnologia (EJPRD/0001/2020), Consultant of: Received payment or honoraria from MSD (Portugal), Neurolite AG, and the European Monitoring Centre for Drugs and Drug Addiction; received support for attending meetings from Janssen (Portugal); participated in advisory boards for Angelini (Portugal) and Janssen (Portugal), Employee of: Vice-President of the Portuguese Society for Psychiatry and Mental Health; Head of the Psychiatry Working Group for the National Board of Medical Examination (GPNA) at the Portuguese Medical Association and Portuguese Ministry of Health

